# Pd supported on carbon containing nickel, nitrogen and sulfur for ethanol electrooxidation

**DOI:** 10.1038/s41598-017-15060-x

**Published:** 2017-11-13

**Authors:** Zi-Rui Yang, Shang-Qing Wang, Jing Wang, Ai-Ju Zhou, Chang-Wei Xu

**Affiliations:** 1 Guangzhou Key Laboratory for Environmentally Functional Materials and Technology, School of Chemistry and Chemical Engineering, Guangzhou University, Guangzhou, 510006 China; 2 Guangzhou Key Laboratory for New Energy and Green Catalysis, Guangzhou University, Guangzhou, 510006 China

## Abstract

Carbon material containing nickel, nitrogen and sulfur (Ni-NSC) has been synthesized using metal-organic frameworks (MOFs) as precursor by annealing treatment with a size from 200 to 300 nm. Pd nanoparticles supported on the Ni-NSC (Pd/Ni-NSC) are used as electrocatalysts for ethanol oxidation in alkaline media. Due to the synergistic effect between Pd and Ni, S, N, free OH radicals can form on the surface of Ni, N and S atoms at lower potentials, which react with CH_3_CO intermediate species on the Pd surface to produce CH_3_COO^−^ and release the active sites. On the other hand, the stronger binding force between Pd and co-doped N and S is responsible for enhancing dispersion and preventing agglomeration of the Pd nanoparticles. The Pd(20 wt%)/Ni-NSC shows better electrochemical performance of ethanol oxidation than the traditional commercial Pd(20 wt%)/C catalyst. Onset potential on the Pd(20 wt%)/Ni-NSC electrode is 36 mV more negative compared with that on the commercial Pd(20 wt%)/C electrode. The Pd(20 wt%)/Ni-NSC in this paper demonstrates to have excellent electrocatalytic properties and is considered as a promising catalyst in alkaline direct ethanol fuel cells.

## Introduction

Fuel cells are widely recognized as very attractive devices to obtain directly electric energy through an electrochemical reaction between anode and cathode^1^. Among the power sources, direct methanol fuel cells (DMFCs) have been extensively investigated and proposed to be used as mobile applications^2^. Compared to methanol, ethanol is a promising alternative fuel with less toxicity. Direct ethanol fuel cells (DEFCs) have been extensive studied by many research groups^3–6^. As emerging technologies, DEFCs have many challenges that need to be addressed. One of the major hurdles of the DEFCs is the ethanol electrooxidation reaction which exists slow sluggish kinetics due to the twelve-electron transfer process^7,8^. Platinum (Pt) and Pt-based nanoparticles have been generally used as the acceptable anode materials for the electrooxidation of liquid fuels such as methanol and ethanol^9–11^. Unfortunately, high cost and susceptible to poisoning by CO make it practically impossible to be an ideal candidate at a commercial level. Therefore, exploring cost-effective and catalytically active materials is of great interest to study.

Compared with Pt, palladium (Pd) has numerous advantages in the DEFCs^9,10,12–15^. Xu and Shen^9,16^ firstly found that Pd-based catalysts have remarkable activity for ethanol oxidation in alkaline media in 2006. However, it is still necessary to decrease the dosage of Pd and lower the catalyst cost. Lots of methods have been proposed such as modifying Pd particles with transition metal oxides including NiO, MnO_2_, MoO_3_, CeO_2_, Co_3_O_4_, CuO and TiO_2_^10,16–19^. Among such transition metal oxides, NiO nanoparticles can improve the oxidation kinetics and reduce the poisoning of Pd/C, due to the excellent capacity to transport surface lattice oxygen and OH_ads_^10,20,21^. However, general routes to synthesize NiO nanoparticles are usually on the surface of carbon materials, which lead to heterogeneous dispersion and even result in the surface shed off. Thus, it is urgent to choose a template to arrange the growth of transition metal oxides. Recently, porous carbon materials synthesized by directly annealing coordination compounds especially metal-organic frameworks (MOFs) possess ultrahigh surface area and periodic network of highly ordered three-dimensional framework structure, which are highly desirable as stable supports to load precious metals^22–25^.

Here, we used the vanillic thiosemicarbazone ligand (L = C_9_H_10_N_3_O_2_S) including N and S heteroatoms and its coordination compound NiL_2_ as precursors to obtain porous carbon materials containing nickel, nitrogen and sulfur (Ni-NSC), which can be easily synthesized. Through carbonization and doping steps, the Pd nanoparticles supported on the Ni-NSC (Pd/Ni-NSC) can be successfully obtained. The as-prepared porous Pd/Ni-NSC materials will be used as catalysts for ethanol electrooxidation. Zhong *et al*. have studied the PdNi/C nanoparticles by X-ray diffraction (XRD) and X-ray photoelectron spectroscopy (XPS) and revealed the presence of metallic Ni and the oxide phases NiO, Ni(OH)_2_, NiOOH in the catalyst matrix^21^. So PdNi materials have been used as electrocatalysts for the ethanol oxidation reaction in alkaline media^14,26,27^. Niu *et al*.^28^ have reported that small palladium nanoparticles (Pd NPs) inside sulfur-doped carbon microsphere (S-CMS) show both high electrocatalytic activity and long durability for methanol oxidation reaction (MOR) in the DMFCs. Wei *et al*. have studied the interaction between nitrogen in the carbon nanotubes (CNTs) and Pd in the catalysts^29^. The nitrogen-doped CNTs-supported Pd catalysts exhibit superior electrochemical activity for ethanol oxidation relative to the pristine CNTs. Zhang *et al*.^30^ have reported that the introduced nitrogen and sulfur co-doping could generate abundant active sites on the graphene surface supported Pd nanoparticles (Pd/NS-rGO) and the Pd/NS-rGO catalyst also reveals superior anti-poisoning ability and amazing stability.

## Results and Discussion

**Figure 1:**
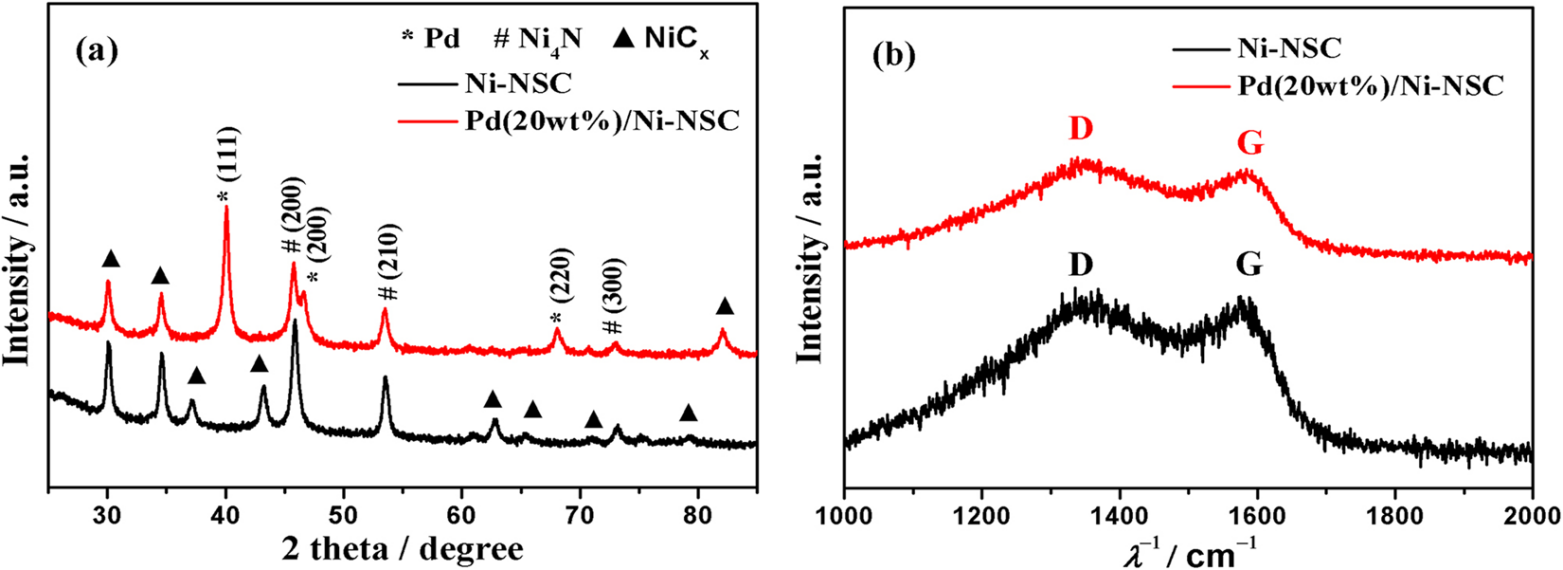
(**a**) XRD patterns and (**b**) Raman spectra for Ni-NSC and Pd(20 wt%)/Ni-NSC.

XRD patterns for the Ni-NSC and Pd(20 wt%)/Ni-NSC were presented in Fig. 1a. All the characteristic peaks could be well observed corresponding to the standard patterns of NiC_x_, Ni_4_N and Pd (JCPDS card No. 45–0979, 36–1300 and 65–2867). The diffraction peaks around 48.6, 55.0 and 76.0° were assigned to the (200), (210) and (300) facets of the cubic crystallite Ni_4_N. The strong diffraction peaks at the Bragg angles of 40.1, 46.6, 68.1, 82.1 and 86.6° correspond to the (111), (200), (220), (311) and (222) facets of the face-centered cubic crystallite Pd. The diffraction peaks associated with Ni_4_N derived from Ni compound in the Pd(20 wt%)/Ni-NSC composite show weaker peaks in comparison with the signal of Ni-NSC unloading Pd. Raman measurements of Ni-NSC and Pd(20 wt%)/Ni-NSC further confirm the structural composition as shown in Fig. 1b. The characteristic D and G bands of graphitic carbon can be seen at 1340 and 1589 cm^−1^ in both two materials, which could be assigned to the defective and graphitic structure of the carbon materials^31^. D band arises from disordered sp^3^ defect sites and is also associated with defects and disorder in carbon structure^32^. The degree of graphitization of carbon materials can be quantified by the intensity ratio of D to G bands. In this case, those defects will in turn act as active sites and reinforce the interaction between Pd particles and support, and thus increase the catalysts loading and particles dispersion. The *I*_D_/*I*_G_ ratio of Pd(20 wt%)/Ni-NSC is 1.05, which is smaller than that of Ni-NSC(1.11). It may be attributable to the supporting Pd, which can decrease defect sites and disorder in carbon structure.

**Figure 2:**
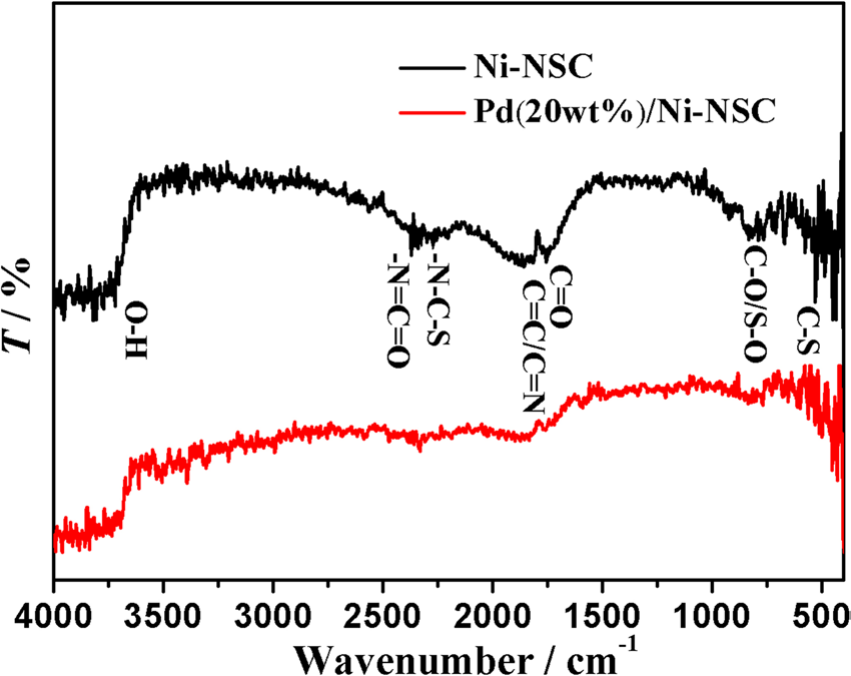
FTIR spectra of Ni-NSC and Pd(20 wt%)/Ni-NSC.

The Fourier transform infrared (FTIR) spectra in Fig. 2 confirm various surface groups of Ni-NSC. The peaks at 3570 cm^−1^ in the spectra should be considered as the O-H stretching vibration. The peak at 1755 cm^−1^ is attributed to the C=C and C=N stretching vibration in the conjugated structure^33^. In addition, two obvious peaks at 2414 and 2389 cm^−1^ in the spectrum of Ni-NSC should be ascribed to the –N=C=O and -N-C-S stretching vibration, respectively. It indicates that the N and S atoms have been doped to the Ni-NSC. The absorbance from 970 to 1200 cm^−1^ is attributed to the stretching vibrations of the C-O-C and C-S-C bond^34^. Besides, the bending vibration of the C-S bond also shows a weak peak at 646 cm^−1^, which confirms the successful doping of sulfur element. Moreover, surface groups of Pd(20 wt%)/Ni-NSC compared with that of Ni-NSC in FTIR spectra have a weaker peak, which is due to Pd nanoparticles growing on the Ni-NSC surface and weakening the absorption intensity.

**Figure 3:**
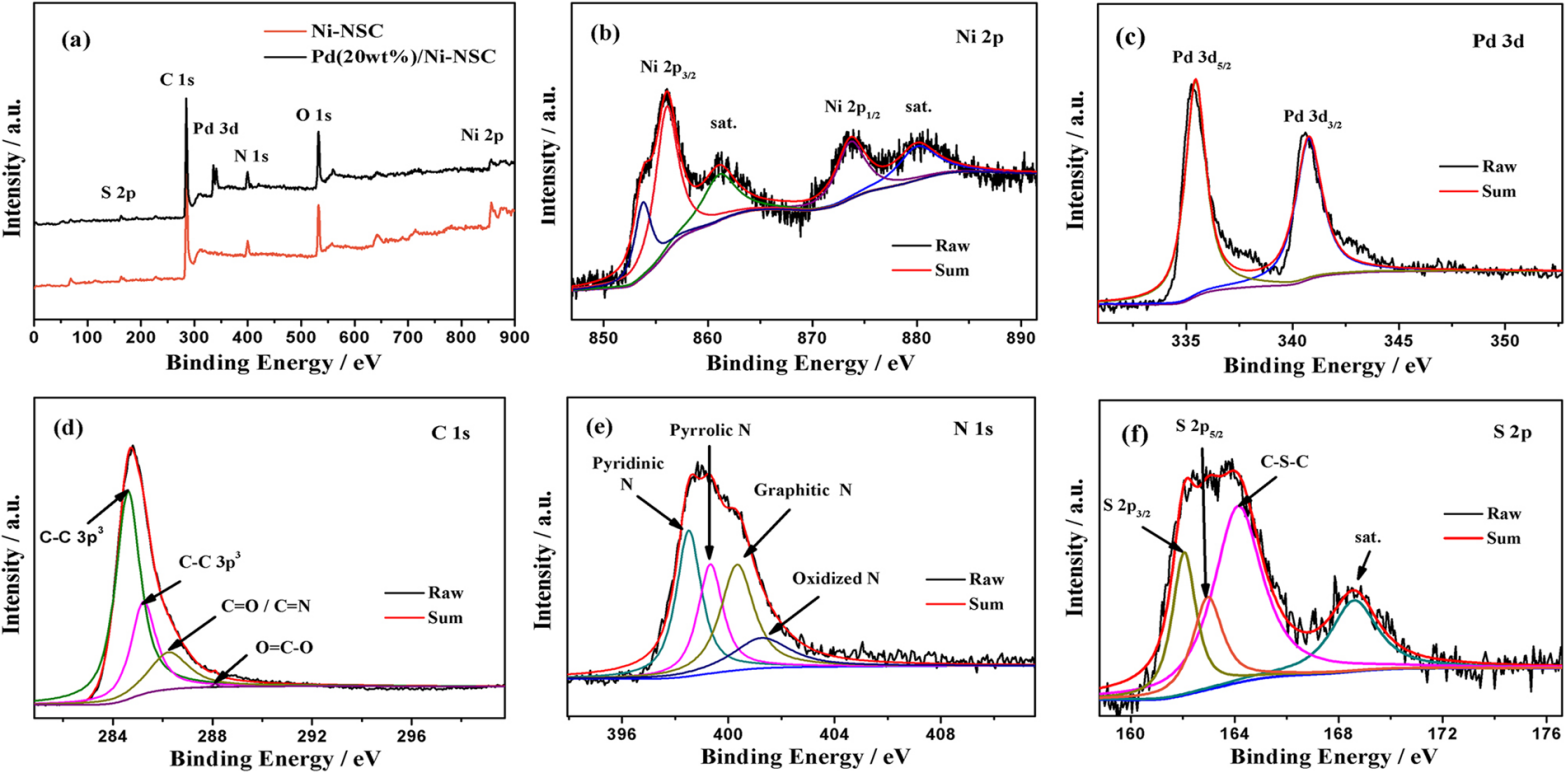
XPS spectra of (**a**) overall for Ni-NSC and Pd(20 wt%)/Ni-NSC; (**b**) Ni 2p, (**c**) Pd 3d, (**d**) C 1 s, (**e**) N 1 s and (**f**) S 2p for Pd(20 wt%)/Ni-NSC.

Chemical bonding states in the Pd(20 wt%)/Ni-NSC were analyzed by XPS (Fig. 3). From the compared survey of Ni-NSC and Pd(20 wt%)/Ni-NSC shown in Fig. 3a, the peaks are corresponding to existence of Pd 3d, Ni 2p, C 1 s, N 1 s and S 2p, respectively. Ni 2p spectrum presents a complicated structure with two strong satellite peaks adjacent to the main peaks as shown in Fig. 3b, which is due to multi-electron excitation. The presence of multiple-valence nickel element in the Pd(20 wt%)/Ni-NSC is apt to occur redox reaction due to electron transfer. The binding energy values of XPS spectrum of Pd 3d display a doublet that is composed of a low-energy band (Pd 3d_5/2_) and a high-energy band (Pd 3d_3/2_) at 335.4 and 340.7 eV (Fig. 3c), which are similar to the typical Pd^0^ specie as previous report^35^. These above data show that Pd specie attached to the surface of Ni-NSC exists in the form of Pd^0^. From the high resolution peak split in Fig. 3d, the C 1 s spectrum is deconvoluted into four peaks, sp^2^-hybridized graphite-like carbon C=C at 284.6 eV, sp^3^-hybridized diamond-like carbon C-C at 285.2 eV, C−O−C/C-N bond at 286.2 eV and O=C–O bond at 288.1 eV^36^. The high resolution N 1 s spectrum in Fig. 3e can be resolved into four sub-peaks due to the spin–orbit coupling, including pyridinic-N (398.5 eV), pyrrolic-N (399.7 eV), graphitic-N (400.7 eV) and pyridine-N-oxide groups (401.7 eV)^37^. Besides, the S 2p spectrum can be deconvoluted to one pair of spin-orbit doublet at 161.0 (S 2p_3/2_) and 162.9 eV (S 2p_5/2_) in Fig. 3f, indicating the existence of metal sulfide and formation of C-S-C at 164.1 eV^37^. These results of the existence of mentioned-above chemical bonds illustrate that Ni, N and S atoms are doped well into the carbon material to form Ni-NSC, in which Pd nanoparticles are inserted.

**Figure 4:**
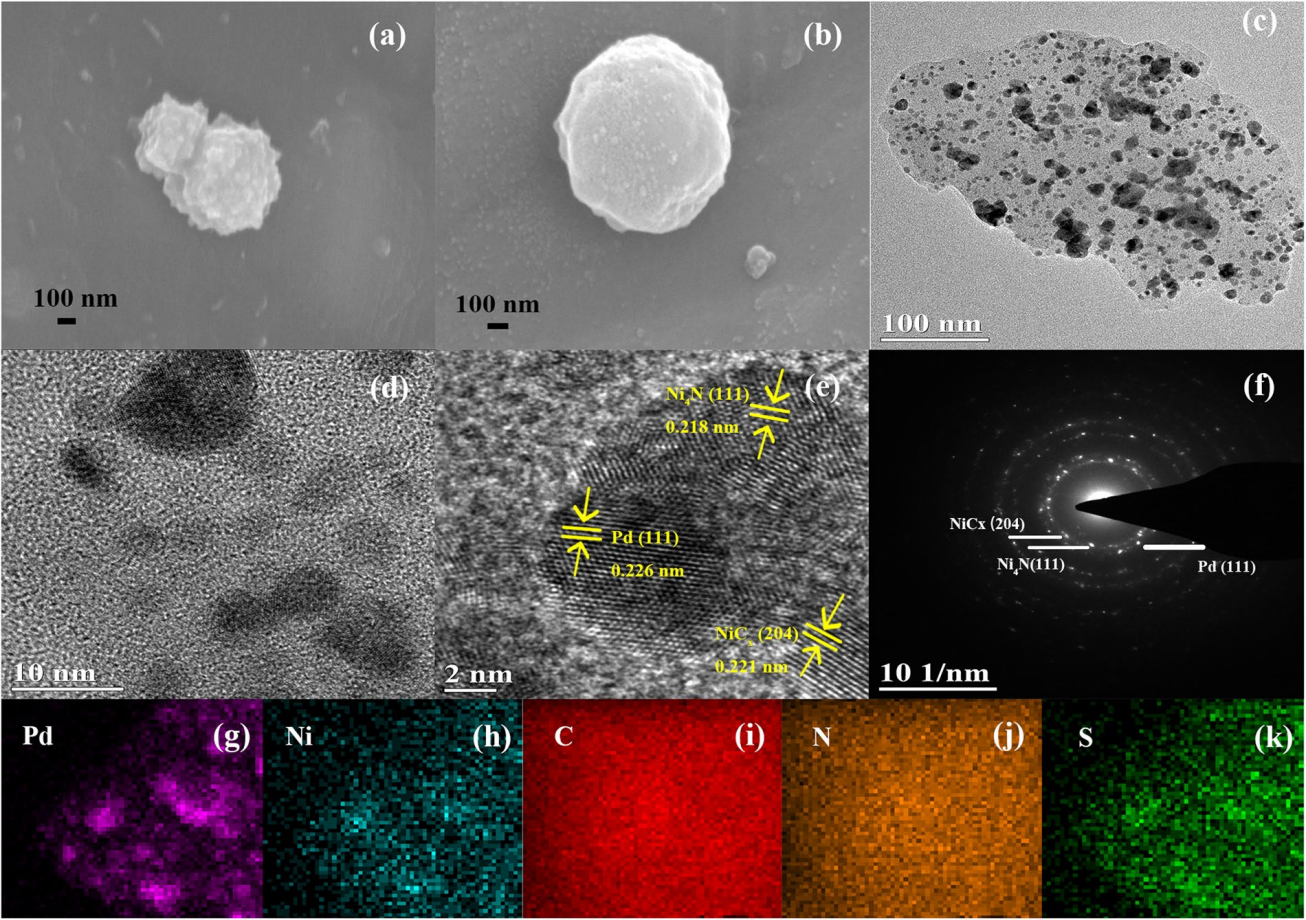
(**a**,**b**) SEM images, (**c**–**e**) TEM images, (**f**) SAED image and (**g**–**k**) element mapping images for Pd(20 wt%)/Ni-NSC.

The as-synthesized microporous Ni-NSC and Pd(20 wt%)/Ni-NSC materials were characterized using scanning electron microscopy (SEM) (Fig. 4a,b). It is found that the size of the Ni-NSC and Pd(20 wt%)/Ni-NSC particles is both ranged from 200 to 300 nm. The typical transmission electron microscopy (TEM) and high-resolution TEM (HRTEM) images for Pd(20 wt%)/Ni-NSC are shown in Fig. 4c,d. It is clearly observed that deep shadow particles on microporous Ni-NSC known as Pd nanoparticles exhibit a spherical-like shape with well-dispersion. As shown in Fig. 4e, it clearly reveals a lattice spacing of 0.218 and 0.221 nm, which correspond to the distance of the (111) crystal plane of Ni_4_N and the (204) one of NiC_x_. Besides, one lattice fringe measured from the image can be found around 0.226 nm, which corresponds to the distance of the (111) crystal plane of Pd. Figure 4f is the selected-area electron diffraction (SAED) pattern of Pd(20 wt%)/Ni-NSC, where a series of well-defined rings can be assigned to Ni_4_N(111), NiC_x_(204) and Pd(111). Elemental mapping results (Fig. 4g–k) confirm the presence of Pd, Ni, C, N and S components in Pd(20 wt%)/Ni-NSC. All the results show that the elements of Pd, Ni, C, N and S can be observed, indicating their coexistence in the catalysts.

**Figure 5:**
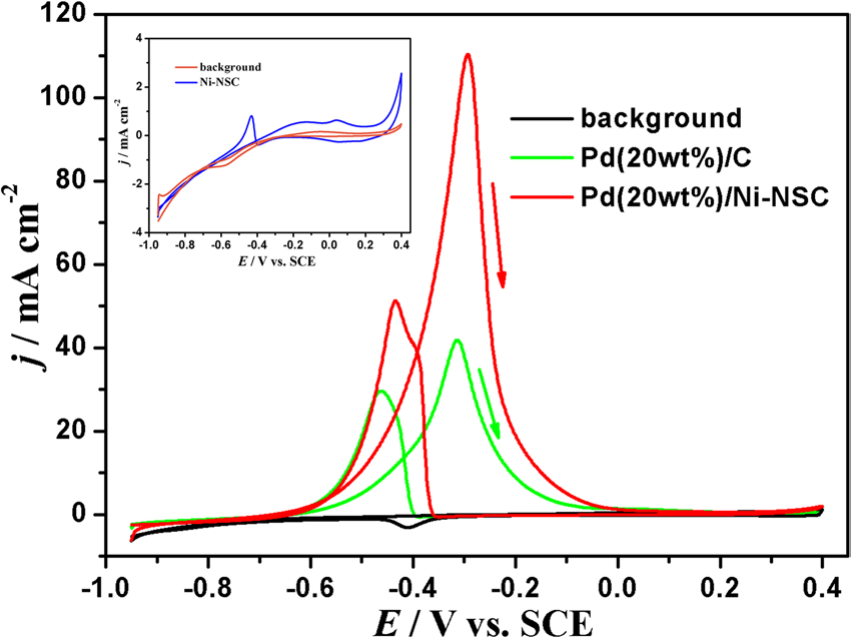
CV curves in 1.0 mol L^−1^ ethanol and 1.0 mol L^−1^ KOH with a sweep rate of 50 mV s^−1^ on Pd(20 wt%)/Ni-NSC and commercial Pd(20 wt%)/C electrodes. (The inset figure is CV curve in 1.0 mol L^−1^ ethanol and 1.0 mol L^−1^ KOH with a sweep rate of 50 mV s^−1^ on Ni-NSC electrode).

For the purpose of comparison, commercial Pd(20 wt%)/C from Sigma-Aldrich was also investigated. Cyclic voltammetry (CV) measurements for ethanol electrooxidation on the Pd(20 wt%)/Ni-NSC and commercial Pd(20 wt%)/C electrodes were carried out in 1.0 mol L^−1^ KOH containing 1.0 mol L^−1^ ethanol solution with a sweep rate of 50 mV s^−1^ at a Pd loading of 0.10 mg cm^−2^ to evaluate their electrocatalytical activity as shown in Fig. 5. The background is the CV on the Pd(20 wt%)/Ni-NSC electrode measured in a nitrogen-saturated 1.0 mol L^−1^ KOH solution without ethanol. The magnitude of oxidation current on the forward scan indicates the activity of electrocatalyst for ethanol oxidation, which is corresponding to the oxidation of freshly chemisorbed species from ethanol adsorption. As shown in the inset figure of Fig. 5, the Ni-NSC shows poor performance for ethanol oxidation. Compared with the CV in the absence of ethanol, an ethanol oxidation peak can be clearly observed in the CV curve on the Pd(20 wt%)/Ni-NSC electrode in the presence of 1.0 mol L^−1^ ethanol. It is obviously that the activity of ethanol oxidation on the Pd(20 wt%)/Ni-NSC electrode is much higher than that on the commercial Pd(20 wt%)/C electrode. The onset potential (*E*_s_) is −0.628 V on the Pd(20 wt%)/Ni-NSC electrode, which is 36 mV more negative compared with that on the commercial Pd(20 wt%)/C electrode (−0.592 V). The lower value of *E*_s_ shows easier electrooxidation of ethanol. The current for ethanol electrooxidation on the Pd(20 wt%)/Ni-NSC electrode begins to rise much more sharply at more negative potential than that on the commercial Pd(20 wt%)/C electrode. It demonstrates that ethanol can be more easily electrochemically oxidized on the Pd(20 wt%)/Ni-NSC electrode than that on the commercial Pd(20 wt%)/C electrode. The peak current density (*j*_*p*_) is 110.3 mA cm^−2^ (−0.293 V) on the Pd(20 wt%)/Ni-NSC electrode, which is 2.6 times as high as that on the commercial Pd(20 wt%)/C electrode, which is 41.8 mA cm^−2^ (−0.316 V). Current density at the potential of 0.3 V (*j*_−0.3V_) is 108.1 mA cm^−2^ on the Pd(20 wt%)/Ni-NSC electrode, which is 2.8 times as high as that on the commercial Pd(20 wt%)/C electrode, which is 38.0 mA cm^−2^.

**Figure 6:**
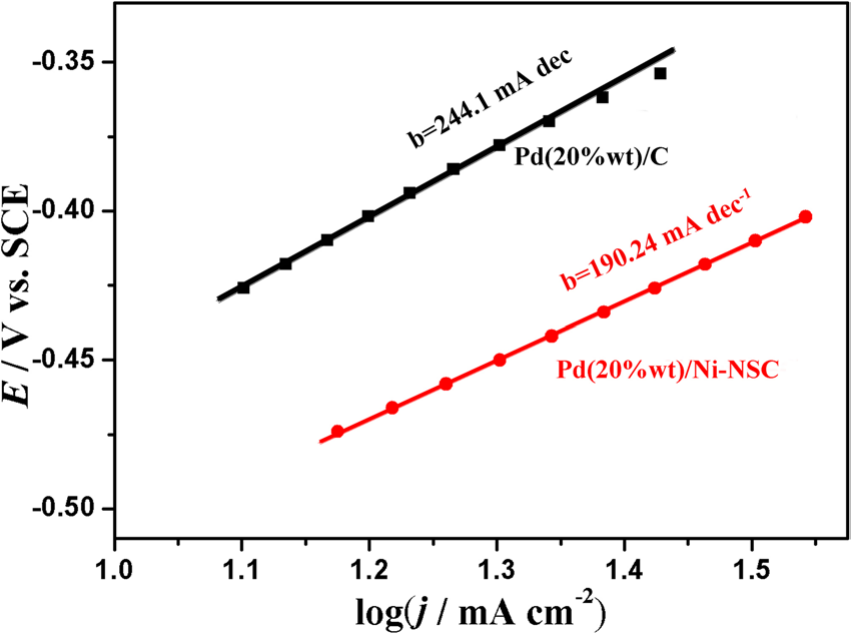
Tafel plots in 1.0 mol L^−1^ ethanol and 1.0 mol L^−1^ KOH with a sweep rate of 2 mV s^−1^ on Pd(20 wt%)/Ni-NSC and commercial Pd(20 wt%)/C electrodes.

In addition, the compared Tafel plots of ethanol electrooxidation, calculated from the quasi-steady-state curve obtained in 1.0 mol L^−1^ KOH containing 1.0 mol L^−1^ ethanol solution with a sweep rate of 2 mV s^-1^ at a Pd loading of 0.10 mg cm^−2^, show linear region with the respective Tafel slope of 190.24 mV dec^-1^ on the Pd(20 wt%)/Ni-NSC electrode and 244.21 mV dec^−1^ on the commercial Pd(20 wt%)/C electrode as shown in Fig. 6. The Tafel value on the Pd(20 wt%)/Ni-NSC electrode is lower than that on the commercial Pd(20 wt%)/C electrode, which shows that ethanol oxidation occurs favourably on the Pd(20 wt%)/Ni-NSC electrode. In ethanol electrooxidation controlled by the adsorption of OH_ads_, the expected Tafel slope is 120 mV dec^-1^ and a positive deviation may be explained by the formation of an inactive oxide layer on the palladium surface and by the 2D structure of the catalytic layer^38^.

**Figure 7:**
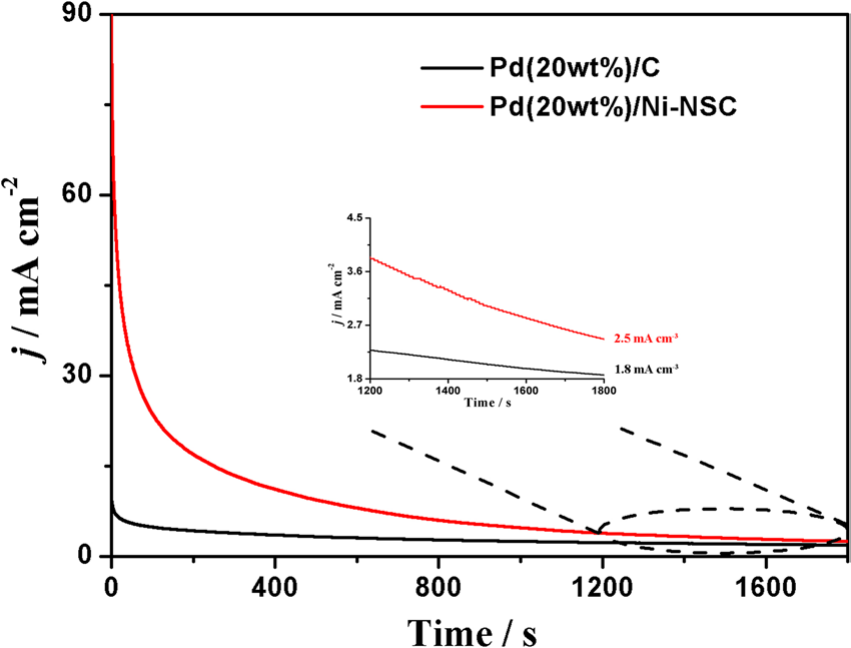
Chronoamperomety curves in 1.0 mol L^−1^ ethanol and 1.0 mol L^−1^ KOH at −0.3 V on Pd(20 wt%)/Ni-NSC and commercial Pd(20 wt%)/C electrodes.

To further evaluate the electrocatalyst stability, the method of chronoamperometry for ethanol oxidation in 1.0 mol L^−1^ KOH solution containing 1.0 mol L^−1^ ethanol at −0.3 V was taken as shown in Fig. 7. It is well-known that the intermediate species such as CO-like species during alcohol oxidation will block the electrode surface to poison the catalyst, then the current of alcohol oxidation will decrease^39^. Nevertheless, at the end of the test, the oxidation current density is 2.5 mA cm^−2^ on the Pd(20 wt%)/Ni-NSC electrode which is larger than that on the commercial Pd(20 wt%)/C electrode (1.8 mA cm^−2^). The good stability of Pd/Ni-NSC catalyst could be relevant with well-dispersed Pd particles, the stronger binding force between Pd and co-doped N and S to inhibit agglomeration of particles^29,30^. It is generally considered that co-doped two-types N and S are responsible for the interaction with metal catalysts and prevent them from aggregation together^30,40^.

The ethanol electrooxidation on Pd-based electrocatalysts is as following step^26,30^:

1 $${\rm{Pd}}+{{\rm{OH}}}^{-}\leftrightarrow {\rm{Pd}}-{{\rm{OH}}}_{{\rm{ads}}}+{\rm{e}}$$

2 $${\rm{Pd}}+{{\rm{CH}}}_{{\rm{3}}}{{\rm{CH}}}_{2}{\rm{OH}}\leftrightarrow {\rm{Pd}}-{({{\rm{CH}}}_{{\rm{3}}}{{\rm{CH}}}_{2}{\rm{OH}})}_{{\rm{ads}}}$$

3 $${\rm{Pd}}-{({{\rm{CH}}}_{{\rm{3}}}{{\rm{CH}}}_{2}{\rm{OH}})}_{{\rm{ads}}}+{{\rm{3OH}}}^{-}\leftrightarrow {\rm{Pd}}-{({{\rm{CH}}}_{{\rm{3}}}{\rm{CO}})}_{{\rm{ads}}}+{{\rm{3H}}}_{{\rm{2}}}{\rm{O}}+3{\rm{e}}$$

4 $${\rm{Pd}}-{({{\rm{CH}}}_{{\rm{3}}}{\rm{CO}})}_{{\rm{ads}}}+{\rm{Pd}}-{{\rm{OH}}}_{{\rm{ads}}}+{{\rm{OH}}}^{-}\leftrightarrow {\rm{2Pd}}+{{\rm{CH}}}_{3}{{\rm{COO}}}^{-}+{{\rm{H}}}_{{\rm{2}}}{\rm{O}}$$

The reaction (4) is the rate-determining step.

The (CH_3_CO)_ads_ is adsorbed onto the surface of the Pd and it can block the active sites and slows the reaction kinetics. The ethanol oxidation on the Pd/NS-rGO may be explained as a bi-functional mechanism^10,41^. Density functional theory calculations reveal that the improved activity and stability stems from the promoted production of free OH radicals (OH_ads_, on Ni active sites) which facilitate the oxidative removal of carbonaceous poison and combination with CH_3_CO radicals on adjacent Pd active sites^14^. The electron-accepting N and S species can impart a relatively high positive charge density on neighboring OH^−^ in the alkaline media, the OH_ads_ species can be more easy formed on the surface of N or S atoms than that on the surface of Pd at lower potentials as following reactions.

5 $${\rm{N}}({\rm{S}})+{{\rm{OH}}}^{-}\leftrightarrow {\rm{N}}({\rm{S}})-{{\rm{OH}}}_{{\rm{ads}}}+{\rm{e}}$$

6 $${\rm{Pd}}-{({{\rm{CH}}}_{{\rm{3}}}{\rm{CO}})}_{{\rm{ads}}}+{\rm{N}}({\rm{S}})-{{\rm{OH}}}_{{\rm{ads}}}+{{\rm{OH}}}^{-}\leftrightarrow {\rm{Pd}}+{\rm{N}}({\rm{S}})+{{\rm{CH}}}_{3}{{\rm{COO}}}^{-}+{{\rm{H}}}_{2}{\rm{O}}$$

The reaction (6) happens more easily than reaction (4). Pd acts as main catalyst for catalysing the dehydrogenation of ethanol during the oxidation reaction and free OH radicals (OH_ads_) can form on the N or S atoms surface at lower potentials. These oxygen containing species react with (CH_3_CO)_ads_ intermediate species on the Pd surface to produce CH_3_COO^−^ and release the active sites. On the other hand, the introduction of N and S atoms significantly changes the electronic structure of carbon materials and the supported Pd nanoparticle. Therefore, due to the electrondonating effects of N and S atoms, the electron cloud density of Pd may increase, which can stabilize Pd, and the N and S groups impart a basic nature to the carbon surface and bind strongly to Pd, enhancing the Pd nanoparticls dispersion and preventing agglomeration of the Pd particles, thereby improving the electrochemical activity and stability of the Pd-based catalysts^29,42^.

In summary, highly active Pd/Ni-NSC materials obtained by heating method and chemical reduction from vanillic thiosemicarbazone ligand (L = C_9_H_10_N_3_O_2_S) and its coordination compound NiL_2_ as precursors to obtain porous carbon materials have been successfully developed and demonstrated as excellent catalysts for efficient ethanol electrooxidation in alkaline media. Thanks to the synergistic effect between Pd and Ni, S, N, free OH radicals can form on the surface of Ni, N and S atoms at lower potentials, which react with (CH_3_CO)_ads_ intermediate species on the Pd surface to produce CH_3_COO^−^ and release the active sites. On the other hand, the electrondonating effects of N and S atoms enhance dispersion and prevent agglomeration of the Pd nanoparticls. The Pd/Ni-NSC shows extraordinary catalytic activity and stability for electrochemical oxidation of ethanol, more competitive than those of the traditional commercial Pd/C catalyst. This work provides new insights into using transition metal oxide and renewable carbon materials as high performance electrocatalyst for alkaline DEFCs.

## Methods

### Materials synthesis

All reagents were of commercially available and analytical grade (AR). All chemicals were purchased from Aladdin and used as received. Vanillin thiosemicarbazone ligands, Ni-vanillic thiosemicarbazone were synthesized according to the literature^40^. Ni-NSC materials were prepared by pyrolyzing the obtained NiL_2_ composite. In a typical procedure, a ceramic boat containing Ni-NSC (500 mg) was placed in a quartz tube, heated under N_2_ atmosphere at a ramping rate of 3 °C min^−1^, and kept at 500 °C for 2 h. Pd/Ni-NSC was prepared by reduction Pd(NH_3_)_4_Cl_2_ on the Ni-NSC powders using an excess 0.01 mol L^−1^ NaBH_4_ solution. The ratio of Pd and Ni-NSC was controlled by stoichiometric calculation with the same weight ratio of commercial Pd/C as 20 to 80.

### Characteriazation

XRD was carried out using a X’Pert powder X-ray diffractometer with Cu K_α_ radiation (*λ* = 0.15418 nm). Raman spectra were obtained using laser confocal micro-Raman spectroscopy (LabRAM HR800, Horiba Jobin Yvon). FTIR spectra were collected using a Tensor27 (Bruker) spectrometer. XPS measurements were performed in an ESCALAB 250 spectrometer under vacuum (about 2 × 10^−9^ mbar). Field-emission SEM images were conducted on a Quanta 400 FEG microscope (FEI Company). TEM images corresponding element energy-dispersive X-ray spectrometer (EDS) were carried out on a JEOL JEM-2010 (JEOL Ltd.). All electrochemical measurements were tested in a three-electrode cell using a CHI 760e electrochemical work station at 25 °C. Solutions were freshly prepared before each experiment. A platinum foil (3.0 cm^2^) was used as counter electrode. All the potentials were measured versus a saturated calomel electrode (SCE, 0.241 V *vs*. NHE) electrode. CV data were recorded between −0.95 to 0.40 V with a scan rate at 50 mV s^−1^.
